# Antibodies as key mediators of protection against *Mycobacterium tuberculosis*

**DOI:** 10.3389/fimmu.2024.1430955

**Published:** 2024-09-02

**Authors:** Qixin Wang, Deepika Nag, Susan L. Baldwin, Rhea N. Coler, Ryan P. McNamara

**Affiliations:** ^1^ Ragon Institute of Massachusetts General Hospital, Massachusetts Institute of Technology, and Harvard University, Cambridge, MA, United States; ^2^ Seattle Children’s Research Institute, Center for Global Infectious Disease Research, Seattle, WA, United States; ^3^ Department of Pediatrics, University of Washington School of Medicine, Seattle, WA, United States; ^4^ Department of Global Health, University of Washington, Seattle, WA, United States

**Keywords:** tuberculosis, antibody, biomarkers, LTBI (latent TB infection), active tuberculosis (ATB), systems immunology, serology

## Abstract

Tuberculosis (TB) is caused by infection with the bacterial pathogen *Mycobacterium tuberculosis* (M.tb) in the respiratory tract. There was an estimated 10.6 million people newly diagnosed with TB, and there were approximately 1.3 million deaths caused by TB in 2022. Although the global prevalence of TB has remained high for decades and is an annual leading cause of death attributed to infectious diseases, only one vaccine, Bacillus Calmette–Guérin (BCG), has been approved so far to prevent/attenuate TB disease. Correlates of protection or immunological mechanisms that are needed to control M.tb remain unknown. The protective role of antibodies after BCG vaccination has also remained largely unclear; however, recent studies have provided evidence for their involvement in protection against disease, as biomarkers for the state of infection, and as potential predictors of outcomes. Interestingly, the antibodies generated post-vaccination with BCG are linked to the activation of innate immune cascades, providing further evidence that antibody effector functions are critical for protection against respiratory pathogens such as M.tb. In this review, we aim to provide current knowledge of antibody application in TB diagnosis, prevention, and treatment. Particularly, this review will focus on 1) The role of antibodies in preventing M.tb infections through preventing Mtb adherence to epithelium, antibody-mediated phagocytosis, and antibody-mediated cellular cytotoxicity; 2) The M.tb-directed antibody response generated after vaccination and how humoral profiles with different glycosylation patterns of these antibodies are linked with protection against the disease state; and 3) How antibody-mediated immunity against M.tb can be further explored as early diagnosis biomarkers and different detection methods to combat the global M.tb burden. Broadening the paradigm of differentiated antibody profiling and antibody-based detection during TB disease progression offers new directions for diagnosis, treatment, and preventative strategies. This approach involves linking the aforementioned humoral responses with the disease state, progression, and clearance.

## Introduction

Tuberculosis (TB), caused by *Mycobacterium tuberculosis* (M.tb), is an infectious disease of the respiratory tract. M.tb is a deadly and highly transmissible airborne pathogen that is passed from person to person through inhalation of respiratory secretions containing viable bacilli. Approximately 10.6 million patients were diagnosed as TB-positive and approximately 1.3 million deaths were attributed to TB in 2022 (1) The only licensed vaccine against TB, Bacillus Calmette–Guérin (BCG), which consists of attenuated *Mycobacterium bovis*, provides a protective effect in newborns and children against disseminated TB but offers less protection against pulmonary TB (2). In a systematic review including 14 reported studies and nearly 3900 children under 16, a 20% reduction in M.tb infection was attributed to BCG vaccination based on an interferon gamma (IFNγ) release assay (3). However, the protection rate may be influenced by infections that occurred prior to vaccination that were either undetectable, asymptomatic, and/or cleared. Due to the global health concern posed by TB and the lack of effective vaccines providing long-term protection against M.tb infection, novel methods for diagnosis, prevention, and therapy for TB are urgently needed.

**Figure 1:**
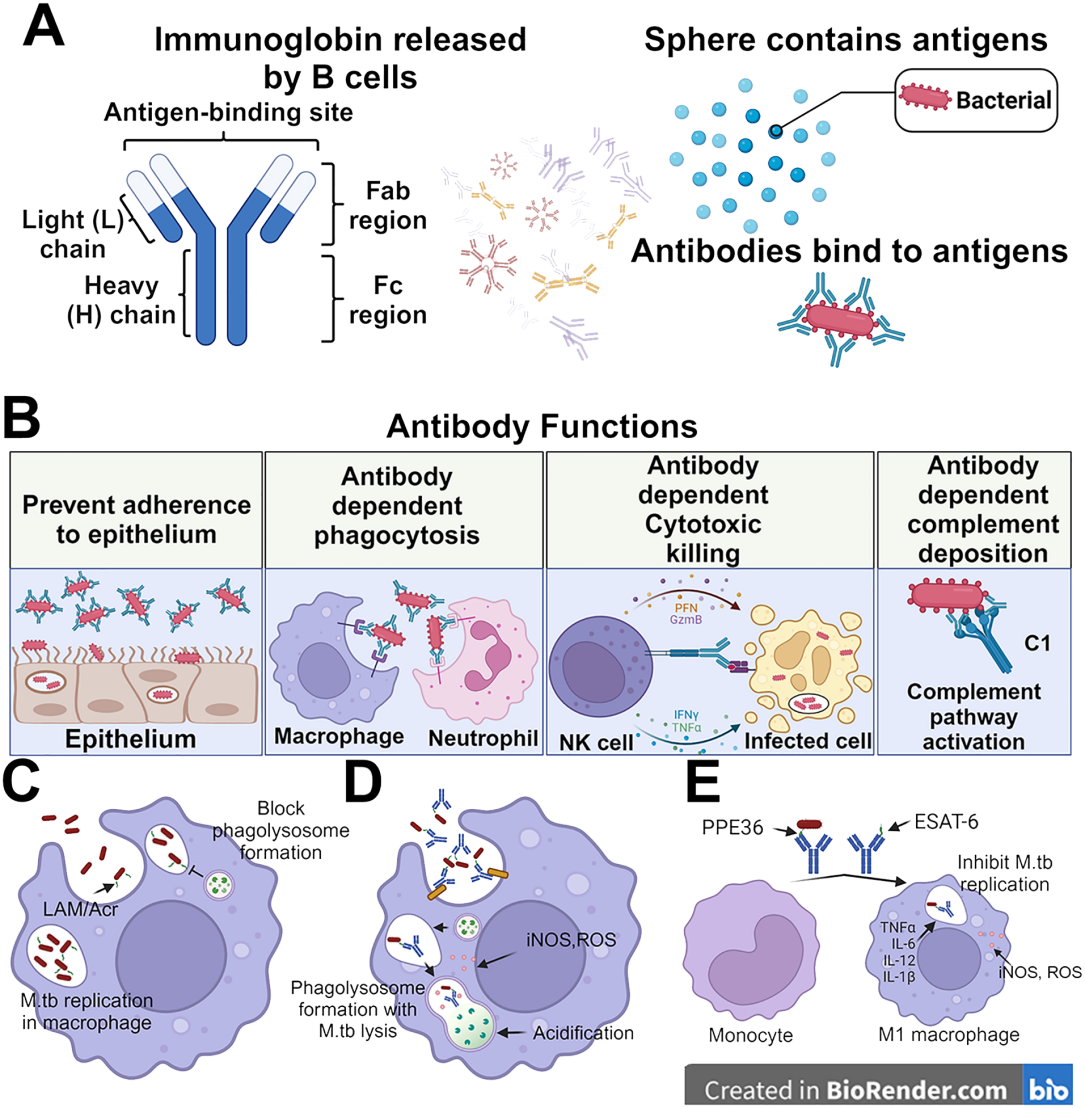
Protective roles for antibodies in M.tb control. **(A)** Antibodies consist of a Fab region which directly binds to target antigens and an Fc region which binds to cell-surfaced expressed receptors. The antibodies can bind to the surface of antigens and mediate different immunity functions. **(B)** Antibodies can leverage both their Fab and Fc domains to protect against M.tb. This can be done through direct binding of the bacteria to prevent adherence to the epithelium, opsinophagocytosis, cellular cytotoxicity activation, and complement deposition. **(C)** The M.tb not bound by the antibodies are able to replicate in the macrophages and inhibit the formation of phagolysosome. **(D)** while the antibody recognition helps the macrophage to lysis the M.tb via lysosome. **(E)** The monocytes also can be differentiated by the anti-PPE36 and anti-ESAT-6 antibodies into M1-polarized macrophages which release inflammatory cytokines to inhibit the self-replication of M.tb in the infected macrophage.

M.tb infection occurs through the respiratory route, typically targeting the alveolar space of the lungs where alveolar macrophages recognize and engulf M.tb (4, 5). The infected alveolar macrophages provide a shelter for M.tb that shields them from extracellular antibodies and complement proteins, which could facilitate bacterial removal. Within 2-8 weeks of initial infection, if M.tb survives the formation of phagolysosomes, which is common in M.tb infection, it undergoes exponential replication and potentially infects other cells such as alveolar epithelium, endothelium, and other leukocytes present in the mucosa such as neutrophils and dendritic cells (5–8). Granulomas, aggregates of these immune cells, are the hallmark of TB and serve as a reservoir of M.tb infection. Mucosal antibodies can theoretically directly block M.tb binding to epithelial cells, while other functional antibody-mediated immune responses can occur in either serum or mucosal sites. These functional antibody responses, including antibody-dependent cellular phagocytosis (ADCP), neutrophil phagocytosis (ADNP), complement deposition (ADCD), and cellular cytotoxicity (ADCC), can play significant roles in identifying and killing infected cells, and help to clear extracellular M.tb, all of which provide additional host immune mechanisms contributing to killing the bacteria at the site of infection preventing dissemination (**Figure 1**).

After decades of investigation, the immune responses and infection process of M.tb have been studied, yet the role of antibodies in preventing infection or disease development remains unclear. One of the challenges that has impeded progress in this area is the variable antibody profiling among TB cohorts, further complicated by the diversity of antibody profiles generated (9–13). We previously demonstrated a positive correlation between IgM, IgA, and IgG levels against LAM, Apa, and PstS1 in serum or bronchoalveolar lavage fluid (BALF) and a decreased M.tb infection rate in non-human primates (NHP) after BCG vaccination (14). Additionally, we found that FcγR binding was higher and ADCC was more activated in patients with latent TB infection (LTBI) compared to those with active TB (ATB). Here, IgG from LTBI patients limited more M.tb surviving in macrophages than IgG from ATB patients (13). These findings suggest that a boosted humoral antibody profile could prevent M.tb infection from its onset, and antibody-mediated functions were critical in controlling the LTBI to ATBI switch in people infected with M.tb.

Advancements in understanding the role of antibodies in preventing M.tb infections, TB development, and the diagnosis of M.tb infections are crucial for developing new clinical methods to prevent the spread of M.tb and the progression of TB disease. Antibodies that protect against M.tb infection could serve as potential standards for vaccine development, while antibody-mediated immunity triggered by M.tb infection could be leveraged for post-infection treatment. Furthermore, antibodies present in the early stages of infection could serve as biomarkers to (i) improve M.tb detection, (ii) classify the disease state, and (iii) predict responses to treatment. Several antibody responses correlated with M.tb infection or after M.tb vaccination, such as opsonophagocytic clearance (ADCP, ADNP), ADCC, and the functional diversity of antibodies against M.tb are discussed (15, 16). In this review, we expand on these concepts and provide a current overview of antibody profiling and its functions in preventing TB development as well as its potential as a diagnostic marker to limit the transmission of M.tb in TB-endemic regions.

## Antibody maturation

Encounters with an antigen begin the process of antibody development and affinity maturation. A B cell receptor (BCR) can bind to an antigen and then signal for its presentation in the form of cleaved peptides to T cells through the major histocompatibility complex 2 (MHC II) on the cell surface. From there, the T cell stimulates B cell activation and proliferation through the interaction of CD40 ligand (CD40L) with CD40 on the B cell surface along with the secretion of various interleukins (ILs) such as IL-2 and IL-5. At this point, the B cell can begin the evolutionary marvel of somatic hypermutation (SHM) (17–19). The Fab domain of an antibody mediates its attachment to an antigen (**Figure 1A**), which is encoded within the heavy (*IgH*) and light (*IgL*) chains of the immunoglobulin genes. After stimulated expression of *IgH* and *IgL*, the B cell undergoes SHM to diversify the Fab’s binding pocket and generate immunoglobins targeting specific antigens. The B cells that produce low-affinity immunoglobin undergo apoptosis (20).

Once an antibody recognizes its targeted antigen, it can mediate a number of downstream processes. One of the most recognized functions is neutralization. Antibodies can directly bind to a region of a pathogen or toxin that is used for cellular entry (**Figure 1B**). While neutralizing antibodies typically target the receptor binding domain (RBD) of pathogen proteins and/or pathogen toxins, they can still exert neutralization at other sites. The primary mechanism of protection by neutralizing antibodies has long been proposed to be through direct steric hindrance of the RBD with its receptor. However, there are clearly other mechanisms by which neutralizing antibodies can inhibit cell entry of a toxin or whole organism/virus into a cell. Currently, no direct evidence proves that neutralizing antibodies exist in preventing M.tb infection.

## Antibody-mediated functions and Mycobacterium tuberculosis infection

### Prevention of adherence to lung epithelium

The abundance and repertoire of antibodies in the mucosa can be distinct from the serum profile within the same individual. The maturation of tissue-resident plasma cells to produce the specific antibody against M.tb antigens is in parallel with the progression of infection, which is common across most infectious diseases (21, 22). The specific mechanisms of antibody subclasses in preventing M.tb adherence to the mucosal surface may also depend on compartmentalization. Currently, although antibodies may play a role in defense against M.tb, there are no known mycobacteria-specific antibodies that can neutralize M.tb or inactivate M.tb extracellularly on their own which results in full sterilizing immunity. Immune responses to M.tb instead likely require many different cell types, representing both cellular and humoral immunity (for a review, see (23)). In BCG-immunized individuals, antibodies directed toward shared antigens should theoretically be able to inhibit or contribute to the prevention of infection. Antibodies specific for heparin-binding hemagglutinin (HBHA) as well as lipoarabinomannan (LAM), both of which are M.tb virulence factors on the surface of the bacilli, show some promise in host defense against M.tb infection. HBHA IgA antibodies at the mucosa, for example, can block M.tb infection of lung epithelial cells (9) which in turn can help prevent dissemination of M.tb from the lungs to other organs. Interestingly, anti-HBHA IgG antibodies were shown to have the opposite effect, instead facilitating M.tb infection (9). Considering the infection route and site of M.tb, mucosal antibodies would be more likely prominent players in preventing the infection state than antibodies circulating in serum. IgA is the most abundant immunoglobins at this interface, although this antibody can also be detected in serum as well (24–26). IgA, specific to several M.tb antigens, can be observed in the pleural fluid in pleural TB patients (27). Nasal-resident IgA also showed a protective effect in inhibiting the M.tb growth and infection with the necessary activation of FcαR (22). In addition to the effective role of IgA, relevant lung epithelial cells express Fc receptors that primarily interact with IgG, providing another potential layer of humoral immunity at this site of M.tb infection.

### Opsonophagocytosis

Intracellular growth inhibition of M.tb following opsonophagocytosis, a process involving antibody-directed phagocytosis by macrophages and neutrophils (24, 26, 28–31), utilizing serum from BCG-immunized humans, has shown a potential protective role for arabinomannan-specific antibodies (32). Furthermore, the opsonophagocytosis process of killed M.tb in the presence of anti-LAM and anti-Acr (a major 16-kD a-crystallin membrane protein (33) donor-derived antibodies also results in phagosomal maturation of M.tb infected macrophages, leading to a cascade of host-mediated microbicidal responses such as the production of nitric oxide and acidification (34) (**Figure 1B**). Our group is currently studying the effects of adjuvants on M.tb vaccine-induced functional antibody responses against M.tb infection including opsonophagocytosis. Generating optimal antibody responses in the context of an immunotherapeutic M.tb vaccine, as an adjunct to drug treatment, could provide additional protection against persistent or recurrent M.tb (24, 26, 28–31).

For IgG subclasses, the Fc-domain can bind to FcγRs on the surface of phagocytic cells. Pro-phagocytic FcγRs include the high-affinity FcγRI (CD64) and the low-affinity FcγRIIA and FcγRIIIB (CD32a and CD16b, respectively). Phagocytosis can also occur through the recognition of complement deposited on an antigen through Fc-C1q. This deposition of complement on an antigen, or on the antibody itself bound to the antigen, can flag the complex for engulfment by phagocytes with complement receptors on their cell surface. Opsonophagocytic antibodies have been linked to protection against other intracellular bacteria as well as M.tb, including *Staphylococcus aureus*, *Escherichia coli*, *Salmonella*, and *Shigella* (35–37).

Regarding opsonophagocytosis, antibody-mediated phagocytosis is followed by phagolysosome fusion to digest antibody-bound complexes/microorganisms. This is enhanced through intracellular FcγR signaling activation (38). Interestingly, it is known that M.tb can inhibit the phagosome-lysosome fusion via a reduced Ca^2+^ pathway. Thus the bacteria can be engulfed by phagocytic cells such as macrophages, but are not killed through canonical opsonophagocytosis signaling (39). This phenomenon was shown to be antigen-specific, for example in the case of M.tb, anti-LAM mediated phagocytosis presented a higher rate of M.tb killing (39). Macrophage activation, especially M1 polarization, occurs during M.tb acute infection, initiates inflammatory cytokine release, and generates oxidative species to eliminate both the intracellular and local extracellular M.tb (40). The M.tb surface protein PPE36 inhibited the M1 polarization and reduced the inflammatory cytokine production from macrophages, which enhanced the survival of M.tb (41, 42). In TB patients, the levels of IgA against PPE36 were found to be significantly higher compared to healthy control, while less elevation was noticed in either IgG or IgM (43). As mentioned above, IgA is the most dominant antibody at the site of M.tb infection, and IgA against PPE36 could inactivate the function of PPE36 and activate the M1 polarization. Another surface protein, Rv1507A, promoted the polarization of M1 macrophages, enhanced the proinflammatory cytokine release of IFN-γ and tumor necrosis factor alpha (TNF-α), and upregulated the macrophage-driven phagocytosis (44). The secreted antigen ESAT-6 also can activate the M1 macrophage polarization, and anti-ESAT-6 immunoglobin helps to inhibit the proliferation of M.tb in the infected macrophages (45–47). Taken together, antibodies targeting either M.tb surface or secreted antigens may augment their immune-evading roles, and shift the humoral response to a more functional opsonophagocytic nature against M.tb. Antibody responses to specific antigens after administration of vaccines that developed against M.tb should be further evaluated to make sure specific categories of antibodies can mediate efficient phagolysosome formation and intracellular removal of M.tb.

In addition to macrophages, neutrophils are also recruited to the site of infection by the released cytokine and chemokines. These neutrophils produce hypochlorous acid, proteases, and cytokines, and can create neutrophil extracellular traps as well as mediate phagocytosis to eliminate extracellular M.tb (48). Our previous study reported that BCG-vaccinated NHP exhibited a higher level of antibody-dependent neutrophil phagocytosis facilitated by anti-LAM immunoglobulin (14). Another study showed that IgG from LTBI patients reduced M.tb burden in infected macrophages more effectively compared to the IgG from ATB patients. On the other hand, ADCP and ADNP were more triggered by antibodies from ATB patients compared to LTBI patients (13). M.tb can block the formation of the phagolysosome which makes the macrophages an ideal environment for M.tb replication and further infection. However, there is limited research on antibody-mediated neutrophil-driven M.tb removal. Understanding how specific antibodies engage neutrophils for phagocytosis and whether phagolysosome formation occurs within these innate cells in the alveoli remains largely unexplored.

### Cellular cytotoxicity killing and natural killer cell activation

Natural killer cells (NK) are major innate lymphoid cells that play a similar role to CD8+ cytotoxic T cells. Unlike their CD8+T cell counterparts, however, NK cells do not express the T cell receptor (TCR) on their surface and can be thought of as more of a pan-cellular innate surveillance cell.

NK cells are activated by antibodies through binding of the FcγRIIIA (CD16a) on the surface (**Figure 1B**). Studies have shown that this binding is correlated with the afucosylation of the Fc-domain in IgG subclasses, predominantly IgG1 and IgG3 (49–52). Downstream activation signals funnel into the secretion of IFN-γ, cellular degranulation, and secretion of other inflammatory/recruiting cytokines such as TNF-α, macrophage inflammatory protein-1β (MIP-1β), and a host of interleukins (IL) such as IL-2, IL-12, and IL-18. The combined result of activation of these signaling networks results in ADCC by the NK cells.

ADCC has been shown to play an instrumental role in combating a wide range of infectious agents including viruses, intracellular bacteria, and protozoa (53–55). The coupled degranulation and recruitment of other inflammatory cells through cytokines allow for the rapid clearance of a localized infection. This same model can be translated to a growing mass of tumor cells whereby ADCC acts to wipe out a growing malignancy (56).

The role of NK cells in protecting against M.tb infection is yet to be fully investigated. Canonical mechanism(s) of elimination of M.tb by NK cells included releasing cytotoxic chemicals (perforin and granulysin) after binding to the bacterium and interacting with macrophages or neutrophils to remove the M.tb indirectly (57, 58). The surface receptors of NK cells, NKp46 and NKp44, can be activated by the M.tb surface antigens galactan and peptidoglycan, and initiate the direct killing of M.tb by NK cells (57, 59). Unlike the opsonized phagocytosis via macrophages and neutrophils, NK cells work differently when interacting with antibody-dependent mechanisms. Directly recognized M.tb antigen by the NKp44-Fc region activates the NK cells, and BCG vaccination enhances the surface expression of NKp44 on NK cells (59). NK cells also recognize and lyse the M.tb-infected macrophages via NKp46 (60).

In terms of antibody-mediated NK cell activation, Irvine et al. showed that the serum antibody targeting LAM in BCG-vaccinated NHP presented limited NK cell activation, while the antibody targeting LAM in lavage fluid showed activation of NK cells (14). The enrichment of activated NK cells in the mucosa can later recognize either the extracellular M.tb or M.tb infected monocytes via NKp44 or TLR‐2 to the peptidoglycan axis (59, 61). Abundance and maturation of NK cells within the pleural fluid are thought to be low, so many of these assays to quantify antibody-dependent effects are in artificial systems (14). That said, there is a growing body of literature implicating the role of NK cells in controlling and removing M.tb within the lower respiratory tract. Whereas some anti-M.tb immunoglobins are able to activate NK cells, including antibodies from the serum of humans immunized with ID93+GLA-SE (62),_further investigation is needed to understand the direct antibody-mediated mechanisms involved in the recognition and elimination of M.tb.

## Antibodies and Mycobacterium tuberculosis vaccines

### Antibody profiling and M.tb vaccine development

The role of antibodies in the control of M.tb has been historically controversial. The BCG vaccine has been licensed and deployed to protect against TB. While efficacy has been strong against severe TB in children and against pulmonary TB in low-endemicity regions, there is low protection against pulmonary TB in TB endemic countries. Moreover, BCG vaccination appears to offer low effectiveness in blunting onward transmission of M.tb (15, 63). The mechanism of protection against TB had been proposed to be mediated through cellular immunity, namely CD8+ and CD4+ T cells. This model was built up in large part due to the intracellular nature of M.tb and not in a cell-free state of the pathogen such as seen with respiratory viruses. However, as our appreciation for the non-neutralizing roles of antibodies has grown, we have had to revisit this long-standing dogma that cellular immunity is the sole means of controlling intracellular pathogens such as M.tb.

Several independent studies have shown that TB vaccines elicit antibody responses that correlate with protection. This includes the M72/AS01_E_ vaccine that protects against disease progression in healthy IFN-γ release assays (IGRA)-positive M.tb exposed individuals (64, 65), pulmonary-delivered BCG in rhesus macaques (66), intravenous-delivered BCG (14), and arabinomannan-protein M.tb Ag85 conjugate vaccine (67). The ID93 vaccine candidate (made up of a fusion of 4 M.tb proteins: Rv2608, Rv3620, Rv1813, and Rv3619) administered with the TLR-4 agonist (GLA-SE), was shown to enhance vaccine-specific IgG1 and IgG3 antibody titers, NK activation, and opsonophagocytosis, in addition to an increased CD4+ T helper 1 (Th1) response (defined by IFN-γ, TNF-α, and IL-2 production from ID93-specific CD4+ T cells) (62). To that end, there is an emerging model in the field of M.tb vaccinations that cellular immunity is not solely responsible for protection against TB; similarly, antibody-mediated protection against TB is not functioning in a vacuum. Instead, both cellular and humoral immunity appear to be working in concert to limit the TB disease state (62).

The protective role of antibodies in TB disease progression has also been supported through the transfer of antibodies from an infected or vaccinated donor. In mice, high-dose IVIG attenuated bacterial growth in the lungs. This protection was lost in athymic mice, further supporting a model where antibodies and cellular immunity work in concert to limit pathogen spread and disease progression (68). Subsequent studies demonstrated that antibodies taken from individuals with high occupational exposure to M.tb could protect against aerosolized challenged mice. The antibodies were reactive towards surface-expressed M.tb antigens, and again, the protection offered by antibodies was dependent on the presence of T cells (69). Mechanistically, the glycosylation status of the Fc domain of the antibody appeared to be linked to bacterial killing. Moreover, the state of TB (active vs. latent) strongly influences the antibody response and activity (13). In ATB patients, higher levels of IgG1 to LAM and PPD, IgA1 against PPD, and IgG3 against groES were identified compared to LTBI patients (70).

### Antibody glycosylation

**Figure 2:**
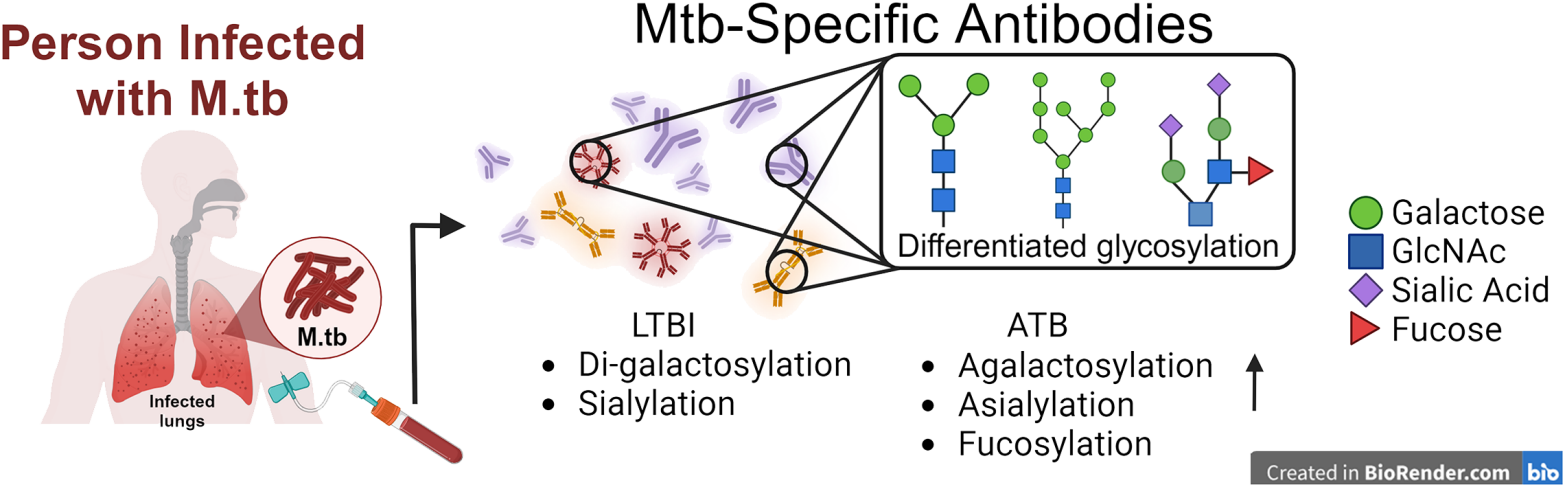
Translating antibody-based readouts novel vaccine platforms. Composite profiling of the total humoral profile can be investigated in the future with differentiated glycosylation signatures in favor of future vaccine designs against TB.

Antibody glycosylation including fucosylation, galactosylation digalactosylation, and sialylation, has been used as biomarkers for differentiating between LTBI and ATB (13) (**Figure 2**). In one study, lower levels of fucose were observed in IgG from LTBI compared to ATB patients (71). Fucosylation/afucosulation status has been shown to be linked to FcγRIIIA binding and downstream signaling (49). Additionally, LTBI patients exhibited increased levels of di-galactosylation and sialylation, along with lower agalactosylation on IgG compared to ATB patients, indicating a heightened inflammatory state in ATB (13, 72–74). Specifically, glycosylation was predominantly observed in Fc regions rather than Fab regions and showed antigen specificity towards PPD and Ag85A (75). Furthermore, differentiated antibody glycosylation distinguishes not only between ATB and LTBI populations, but also identifies differences between ATB and treated ATB groups. Increased sialylation was found in treated ATB patients compared to untreated subjects, while no difference was observed regardless of the treatment (72). Despite extensive research into glycosylated antibodies in various M.tb infection cohorts, little work has been done to characterize the glycosylation status of antibodies following BCG vaccination, the only currently licensed vaccine (76). Notably, antibody glycosylation was only identified after M.tb infection in mice, indicating its potential as a marker of infection (76). Distinct glycosylated antibodies induced by M.tb infections could potentially aid in identifying infected macrophages and promoting phagocytosis or ADCC in cell-surfaced exposed antigens, whereas differentiated glycan removal may attenuate this process (13). RNA/DNA nucleic acid-derived M.tb vaccines expressing proteins that undergo glycosylation within the host cell may detrimentally provide ‘self-glycans’ on the protein masking the epitope needed for lymphocyte recognition and acquired immunity against M.tb (both humoral and cellular), whereas the mycobacterial-derived protein combined with an adjuvant may provide a more robust immune response (77).

## Antibodies as biomarkers for M.tb disease state

M.tb poses formidable challenges due to its ability to establish latent infection, leading to active disease in susceptible individuals (78). Timely and precise identification of TB plays a pivotal role in the efficient handling and containment of the disease. In recent years, there has been a growing interest in utilizing antibodies as biomarkers for assessing the disease state of TB, offering new avenues for improved diagnosis, treatment monitoring, and patient management (13, 72, 75).

### Antibodies as biomarkers to M.tb infection

Upon infection with M.tb, the host immune system mounts a complex response involving various immune cells and immune signaling moieties, including antibodies. It is well established that B cells produce antibodies in response to specific antigens derived from M.tb (79, 80). As mentioned before, antibody treatment significantly decreased the bacterial burden in mice infected with M.tb (81), and IgM responses to BCG were strongly linked with suppressed M.tb survival *in vivo* (14). Studies have also highlighted distinctions in the antibody structure between ATB and LTBI in clinical serum samples (75). This distinction in antibody features in different disease states could be an important biomarker for TB.

Biomarkers are measurable indicators of biological processes, disease states, or responses to therapeutic interventions. In the context of TB, biomarkers represent a significant avenue for enhancing diagnostic precision, evaluating treatment effectiveness, and predicting disease outcomes. Antibodies produced in response to M.tb infection offer valuable insights as biomarkers owing to their specificity and fluctuating levels across the progression of the infection. However, the application of serological tests in TB diagnosis remains debatable due to the poor sensitivity and specificity compared to existing methods (82–84). Ongoing work seeking to standardize antibody signatures as biomarkers for TB will be highly beneficial.

**Figure 3:**
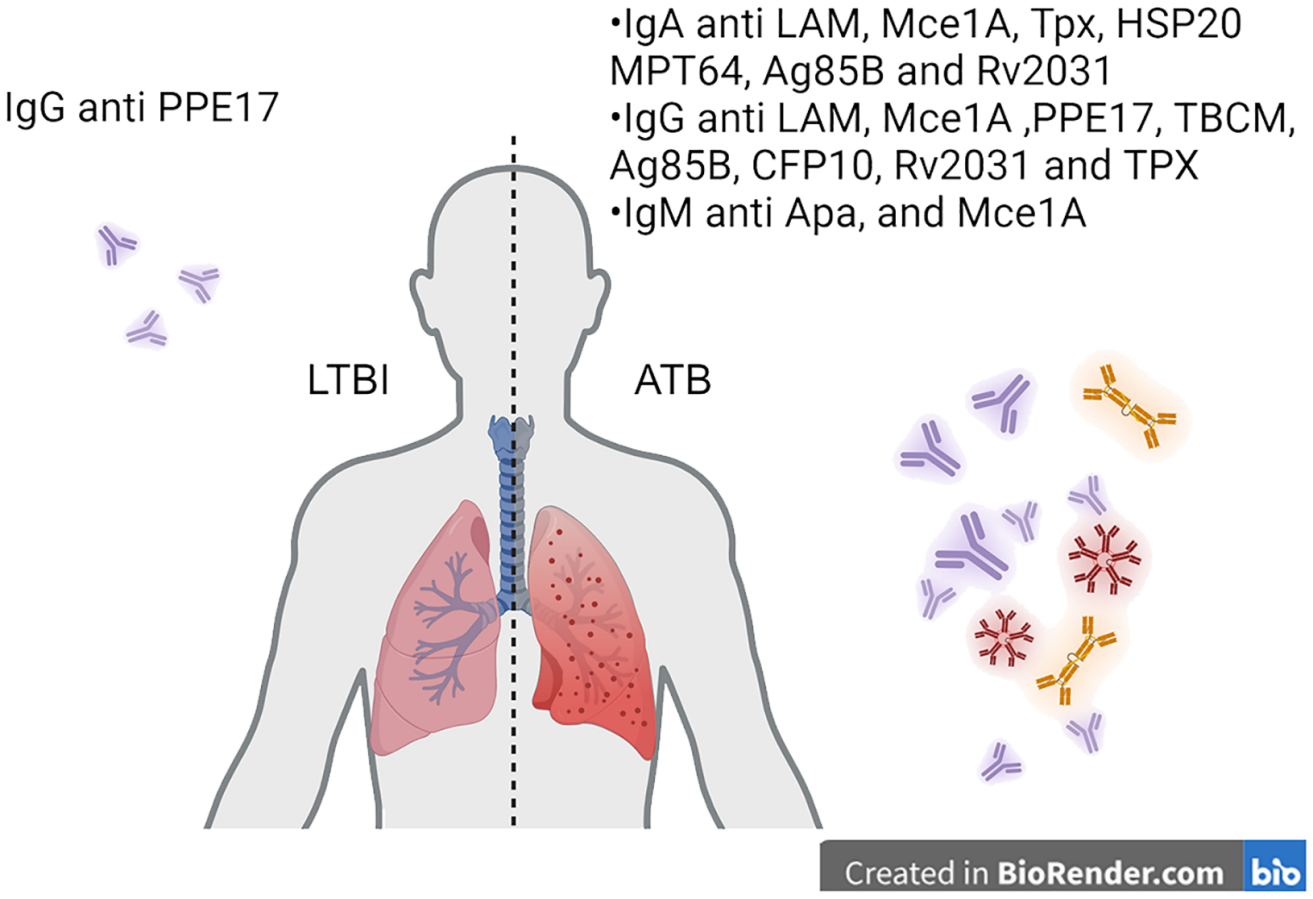
Translating antibody-based readouts to biomarkers of disease states. Antibodies specific to M.tb targets can be analyzed at the isotype and subclass level. Additionally, post-translational modifications can be identified on antigen-specific antibodies to identify signatures of disease states such as latency and active TB.

To date, individual antibodies, or a clustered signature of antibodies against different antigens have both been studied with varied sensitivity and specificity in identifying ATB patients. Several studies have been done to investigate the use of antibodies as biomarkers for identifying TB infection status, as summarized in the tabular form (**Table 1**). The use of single antibody readouts and multivariate analyses have been reported with various sensitivity and specificity readouts as biomarkers. One study using anti-PPE17 IgG showed ~70% sensitivity in identifying an LTBI cohort from non-TB patients (85). IgG and IgA to different M.tb antigens consist of the majority of proposed biomarker candidates in TB diagnosis (80, 83, 87, 106, 107 (86, 87),. Antibody responses to M.tb antigens, specifically IgG against TBCM and CFP-10, and IgG and IgA against Ag85B, can effectively distinguish between active tuberculosis (ATB), latent tuberculosis infection (LTBI), and non-infected individuals, providing a potential biomarker for M.tb infection (86). IgG and IgA levels against the Rv2031 antigen significantly differ among patients with active TB, their household contacts, and non-infected controls (87). An interesting marker for ongoing inflammation and severity of TB disease is IgG4 in humans. In a study including healthy, LTBI, ATB, and treated ATB cohorts, PPD-specific IgG4 as well as HspX and GroEs-specific IgG4 levels were increased in ATB, and lower levels were associated with either LTBI or treated subjects (72). Despite being linked to M.tb control (14), there is limited study on IgM as a biomarker for M.tb infection. This could be due to the inherently low affinity of IgM compared to the affinity selected IgG and IgA (88). Considering the appearance duration of IgM in the humoral antibody profile throughout the infection, IgM may serve as biomarkers for an early, albeit lower-confidence, detection method of M.tb infection. Both IgG and IgA exist both in serum and mucosa and are more likely to play crucial roles in defending against M.tb re-infection or ATB development from LTBI (89). Additionally, it is well known that affinity-matured antibody responses to M.tb are stronger to select antigens such as those on the bacterial cell surface (4, 12, 13, 27, 64–66, 70, 80, 83, 90, 91). Therefore, a panel of IgA and IgG against multiple M.tb antigens could be considered as biomarker candidates to monitor the development of ATB from LTBI patients in TB endemic regions, which the IGRA test cannot identify (86). **Figure 3** demonstrates how the immunoglobulins against antigens could be utilized not only for the detection of active TB but also for distinguishing between ATB and LTBI.

**Table 1:** Antibodies as biomarkers to identify M.tb infection and/or different TB states.

Sample Comparison	Antibody against antigens used	Serological assays defining M.tb infection/state; Ab levels	Sensitivity	Specificity	Reference
LTBI vs NI	IgG against PPE17	ELISA sensitivity to Ag PPE17> ESAT-6:CFP-10 and PPD; ATB/LTBI>NI	69.62%	N/A	(85)
ATB vs NI	IgG against PPE17		94.93%		
ATB vs Control (Non-TB+LTBI)	IgA/IgG against Mce1A	ELISA IgA/IgG anti-Mce1A; ATB>LTBI/NI	59% (IgA), 51% (IgM), 80% (IgG)	77%(IgA), 83% (IgM), and 84% (IgG)	(90)
AFB microscopy positive vs negative	Ig against Rv3881c, Rv0934, Rv0054, Rv3804c, Rv2031c, Rv1886c, Rv0129c, Rv1860	Microbead coating with M.tb antigens; AFB(+) stain>AFB(-) stain	92.20%	74.70%	(91)
	Ig against Rv1980c, Rv3874, Rv0831c, Rv2875, Rv3841, Rv1926c, Rv3875, Rv2878c		93%	79.30%	
ATB vs NI	IgA/IgG against A60	ELISA anti-A60; ATB>NI	31.3%(IgA) and 94% (IgG)	92%(IgA) and 96% (IgG)	(94)
M.tb infection vs Control	IgG against M.TB48	ELISA anti M.TB48; IgG AFB(+) stain>AFB(-) stain	74.1%	97.80%	(113)
Patients with and without culture-confirmed TB	Ig against Ag85B, Ag85A, Ag85C, Rv0934-P38, Rv3881, BfrB, Rv3873, and Rv2878c	MMIA; M.tb infection > NI	90.6%	88.6%	(114)
AFB microscopy positive vs negative	Ig against Rv3881c, Rv0934, Rv2031c, Rv1886c, Rv1860, Rv3874, Rv3875, Rv3804c, Rv3418c, Rv3507, Rv1926c, Rv3874-Rv3875 fusion, Rv2878c, Rv1099, Rv3619, Rv1677, Rv2220, Rv2032, Rv1984c, Rv3873, Rv0054, Rv3841, Rv1566c, Rv2875, Rv0129c, Rv1009, Rv1980c	MMIA; AFB(+) stain>AFB(-) stain	91%	93-99%	(115)
ATB vs healthy	IgG against Rv0310c-E and Rv3425	ELISA; SPPT>SNPT/NI	82.54%	76.92%	(116)
	IgG against Rv1255c-E and Rv3425	ELISA; SNPT>NI			
ATB vs Control (Non-TB+LTBI)	Ig against PstS1, Rv0831c, FbpA, EspB, bfrB, HspX and ssb	LIPS; ATB>NI/LTBI	73.5%	100%	(117)
ATB vs Patient with anti-TB treatment	IgA/IgM anti16kDa	ELISA; ATB>others	90% for both IgA and IgM	95% (IgA) and 90%(IgM)	(99)
ATB vs LTBI	Anti-LAM IgA Anti-TB-LTBI IgG Anti-Tpx IgG Anti-MPT64 IgA	ELISA combination of Abs; ATB>LTBI	N/A	100% accuracy	
ATB vs LTBI/NI	Anti-16 kDa IgA, anti-MPT64 IgA	ELISA; ATB>others	90% for Anti-16 kDa IgA, 90% for anti-MPT64 IgA	95% for Anti-16 kDa IgA, 90% for anti-MPT64 IgA	
ATB vs NI	IgG against LAM	ELISA; ATB>LTBI/NI	71.40%	86.60%	(118)
ATB vs LTBI	IgG against LAM		83.30%	76.40%	
ATB vs NI	IgA anti LAM and HSP20 with IgG anti HSP16.3, LAM, and Tpx		81%	94%	
ATB vs LTBI	IgA anti LAM and Tpx, IgG anti LAM and TPX, IgM anti Apa		81%	91.50%	
ATB vs LTBI vs NI	IgG against TBCM, Ag85B, CFP-10	ELISA; ATB>LTBI>NI	N/A	N/A	(86)
	IgA against Ag85B	ATB>LTBI>NI			
	IgG against Ag85B	ATB>LTBI			
ATB vs Contacts vs Controls (NI)	IgA and IgG against Rv2031	ELISA; ATB>household contacts>NI	N/A	N/A	(87)
ATB vs NI	IgG against Ag85	ELISA; ATB>NI	84.1	85.2%	(119)
	IgG against CFP-10		66	85.2	
	IgG against ESAT-6		64.9	88.9	

ATB, Active TB; LTBI, Latent TB Infection; NI, Not Infected; AFB, Acid-Fast Bacillus; MMIA, Multiplex Microbead Immunoassay; SPPT, Smear-Positive Pulmonary TB; SNPT, Smear-Negative Pulmonary TB; LIPS, Luciferase Immunoprecipitation Systems.

### Detection methods

Several methods are available for detecting M.tb-specific antibodies in patient samples, each with its advantages and limitations. Enzyme-linked immunosorbent assay (ELISA) is a widely used technique that allows for the quantitative measurement of antibody levels in serum, plasma, or other bodily fluids (90, 92, 93). ELISA-based assays offer high sensitivity and specificity and are amenable to high-throughput screening (94). Other approaches, such as multiplex immunoassays (95) and lateral flow assays (96–98), provide rapid and cost-effective alternatives suitable for point-of-care settings where access to laboratory facilities may be limited. Since a combination of M.tb-specific antibodies as biomarkers to evaluate the M.tb infections showed higher sensitivity and specificity than applying individual antibody approaches (99), developing high-throughput methods with multiplexed measurements may not only be more accurate, but also more cost-effective as disease incidence and/or progression biomarkers due to the long experimental duration and high cost of ELISA in detecting individual antibody isotypes/subclasses to discreet antigens, and their body fluid concentration(s). Other than ELISA and Luminex-based measurement, antibody-antigen binding activity measured by surface plasmon resonance (SPR) and total internal reflectance fluorescence (TIRF) microscopy-based biosensors (100, 101) have also been studied. While these technologies are emerging and have unique strengths, they can be limited to the detection of a single antigen or a few antigens at a time. Super-resolution microscopy-based detection methods have also been employed for M.tb protein expression and detection (102). Such a tool, which can utilize antibodies as a detector, has been employed to identify M.tb responses to drug treatments (103).

### Clinical utility

The use of antibodies as biomarkers for TB offers several advantages over traditional diagnostic methods. Antibody-based tests are non-invasive and can be performed on readily accessible samples, such as blood, saliva, or urine, making them suitable for use in diverse settings, including resource-limited areas (95, 96, 104). Moreover, antibody tests have the potential to detect TB infection at an earlier stage than conventional microbiological methods, enabling prompt initiation of treatment and reducing the risk of disease transmission.

Furthermore, longitudinal monitoring of antibody levels can provide valuable insights into treatment response and disease progression. Changes in antibody titers over time may serve as indicators of treatment efficacy, allowing clinicians to tailor therapy regimens and optimize patient care. Additionally, antibody-based biomarkers hold promise for predicting treatment outcomes and identifying individuals at increased risk of disease relapse, thereby informing clinical decision-making, and improving patient outcomes. Biomarkers and antibody signatures of disease/infection are an emerging field of study that warrants further research in the areas of vaccine design, antibody-based therapies, and disease states.

## Challenges and future directions

Although studies have been conducted to understand the antibody differentiation during M.tb infection and TB development, limited vaccines have been developed to protect the adults who are exposed to M.tb, and there is still a knowledge gap in how antibody-mediated immunity controls M.tb infections. The discovery of dynamic antibody profiling at different stages of TB progression and M.tb infection is necessary to design new M.tb vaccines with better efficacy of M.tb removal after infection. Considering the principle of how NK cells participate in eliminating M.tb infected cells is not clear, further investigations are needed to understand the role of antibody-mediated NK cell activation in protecting against M.tb infection and TB development.

Despite their potential, antibody-based biomarkers for TB face several challenges that must be addressed to realize their full clinical utility. Studies show that recognition of M.tb antigens may vary among individuals, leading to the presence of different sets of antigen-specific antibody populations in samples (10), while cross-reactivity among different mycobacterial and nontuberculous mycobacterial species is also observed (105, 106). Variability in antibody responses among individuals, as well as cross-reactivity with other mycobacterial species or non-specific immune responses, may affect the accuracy of antibody-based assays. Standardization of assay protocols, validation in diverse populations, and integration into existing diagnostic algorithms are essential steps toward overcoming these challenges and ensuring the reliability and reproducibility of antibody-based tests for TB.

Furthermore, ongoing research efforts are focused on identifying novel antigen targets and developing innovative assay platforms capable of detecting multiple antibody specificities simultaneously (107). Additionally, the incorporation of antibody-based biomarkers into comprehensive diagnostic strategies, including imaging modalities and nucleic acid amplification tests, holds promise for enhancing the accuracy and efficiency of TB diagnosis and management.

Employment of therapeutic antibodies against TB is an emerging field, as is how to fine-tune humoral responses through vaccination platforms. It is known that vaccine platform and delivery route influence how antibodies leverage both Fab and Fc regions (14, 25, 88, 108–111). Given the emerging role of antibodies as mediators of protection against M.tb, further work characterizing precise mechanisms of protection at various stages within TB disease progress is needed. Such advances can reduce disease incidence and burden.

In conclusion, antibodies stand as hopeful treatments for disease and indicators for evaluating the progression of TB, presenting fresh avenues for enhanced diagnosis, treatment tracking, and patient care. Their unique specificity, ease of access, and fluctuating patterns over the infection timeline render them invaluable instruments for early TB detection, treatment progress monitoring, and prognosis forecasting (**Figure 2**). Through ongoing exploration and creativity, antibody-centric examinations hold the promise to transform TB diagnostics and bolster worldwide endeavors to reduce disease burden.

## Concluding remarks

Antibody responses are not limited to pathogens that exist and/or circulate extracellularly. Moreover, antibody functions often bridge the adaptive and innate arms of the immune response. For example, an affinity-matured IgG or IgA isotype can bind to a target antigen and use its Fc domain to interact with FcRs on the surface of innate immune cells such as NK cells or neutrophils, and initiate inflammatory signaling cascades. This signaling can further recruit additional inflammatory cells to the site of the identified pathogen, helping to clear the infection.

Studies of antibody responses to M.tb have provided significant insight into how antibodies mobilize multiple aspects of the immune system to combat this pathogen. M.tb predominantly resides inside of cells which become constituents of granulomas in the lower respiratory tract. From there, the infection can spread to distant sites in the body including the spleen, kidneys, and brain (112). Antibody responses are not stagnant over time, much like disease states. Due to their adaptability, antibody signatures have been successfully used as biomarkers of disease. This approach has been successfully employed to distinguish between latent and active TB, further supporting the model of humoral fluidity.
